# From FAANG to fork: application of highly annotated genomes to improve farmed animal production

**DOI:** 10.1186/s13059-020-02197-8

**Published:** 2020-11-24

**Authors:** Emily L. Clark, Alan L. Archibald, Hans D. Daetwyler, Martien A. M. Groenen, Peter W. Harrison, Ross D. Houston, Christa Kühn, Sigbjørn Lien, Daniel J. Macqueen, James M. Reecy, Diego Robledo, Mick Watson, Christopher K. Tuggle, Elisabetta Giuffra

**Affiliations:** 1 The Roslin Institute and Royal (Dick) School of Veterinary Studies, The University of Edinburgh, Edinburgh, EH25 9RG UK; 2 Agriculture Victoria, AgriBio Centre for AgriBioscience, Bundoora, Victoria 3083 Australia; 3 School of Applied Systems Biology, La Trobe University, Bundoora, Victoria 3083 Australia; 4 Animal Breeding and Genomics Centre, Wageningen University and Research, 6708 PB Wageningen, The Netherlands; 5 European Molecular Biology Laboratory, European Bioinformatics Institute, Wellcome Genome Campus, Hinxton, Cambridge, CB10 1SD UK; 6 Leibniz Institute for Farm Animal Biology (FBN), Institute of Genome Biology, Genome Physiology Unit, Wilhelm-Stahl-Allee 2, 18196 Dummerstorf, Germany; 7 Faculty of Agricultural and Environmental Sciences, University Rostock, Justus-von-Liebig-Weg 6, 18059 Rostock, Germany; 8 Centre for Integrative Genetics (CIGENE), Department of Animal and Aquacultural Sciences, Norwegian University of Life Sciences, NO-1432 Ås, Norway; 9 Department of Animal Science, Iowa State University, Ames, IA 50011 USA; 10 Université Paris Saclay, INRAE, AgroParisTech, GABI, 78350 Jouy-en-Josas, France

## Introduction

**Fig. 1:**
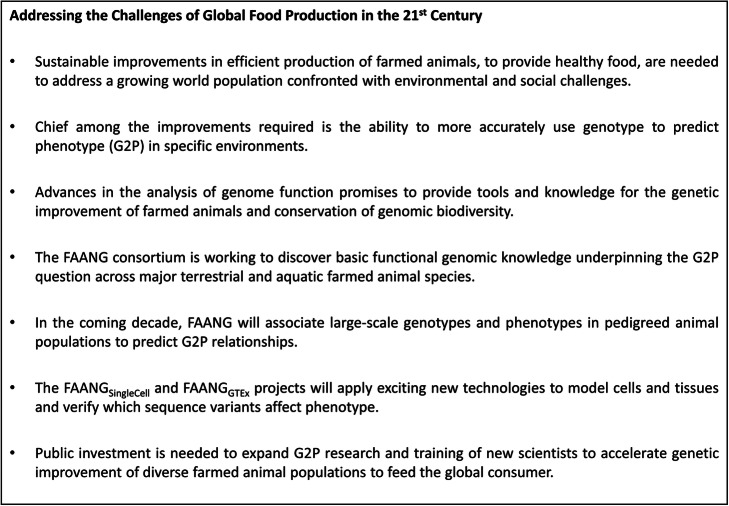
Addressing the challenges of global food production in the 21^st^ Century

The Food and Agriculture Organisation of the United Nations (FAO) reports that by the year 2050 the global human population is likely to reach 9.7 billion, rising to 11.2 billion by 2100 (https://population.un.org/wpp/Publications/Files/Key_Findings_WPP_2015.pdf). This population growth poses several challenges to the global food system, which will need to produce more healthy food using fewer natural resources, reducing the environmental impact, conserving biodiversity and flexibly adjusting to changing societal expectations. Meeting this demand requires environmentally sustainable improvements to farmed animal health and welfare, and of efficiency and diversification (e.g. to include a broader range of locally adapted species) [1]. The changes in breeding strategies and management practises required to meet these goals will need to build on an improved ability to accurately use genotype to predict phenotype in the world’s farmed animal species, both terrestrial and aquatic (Fig. 1).

**Fig. 2:**
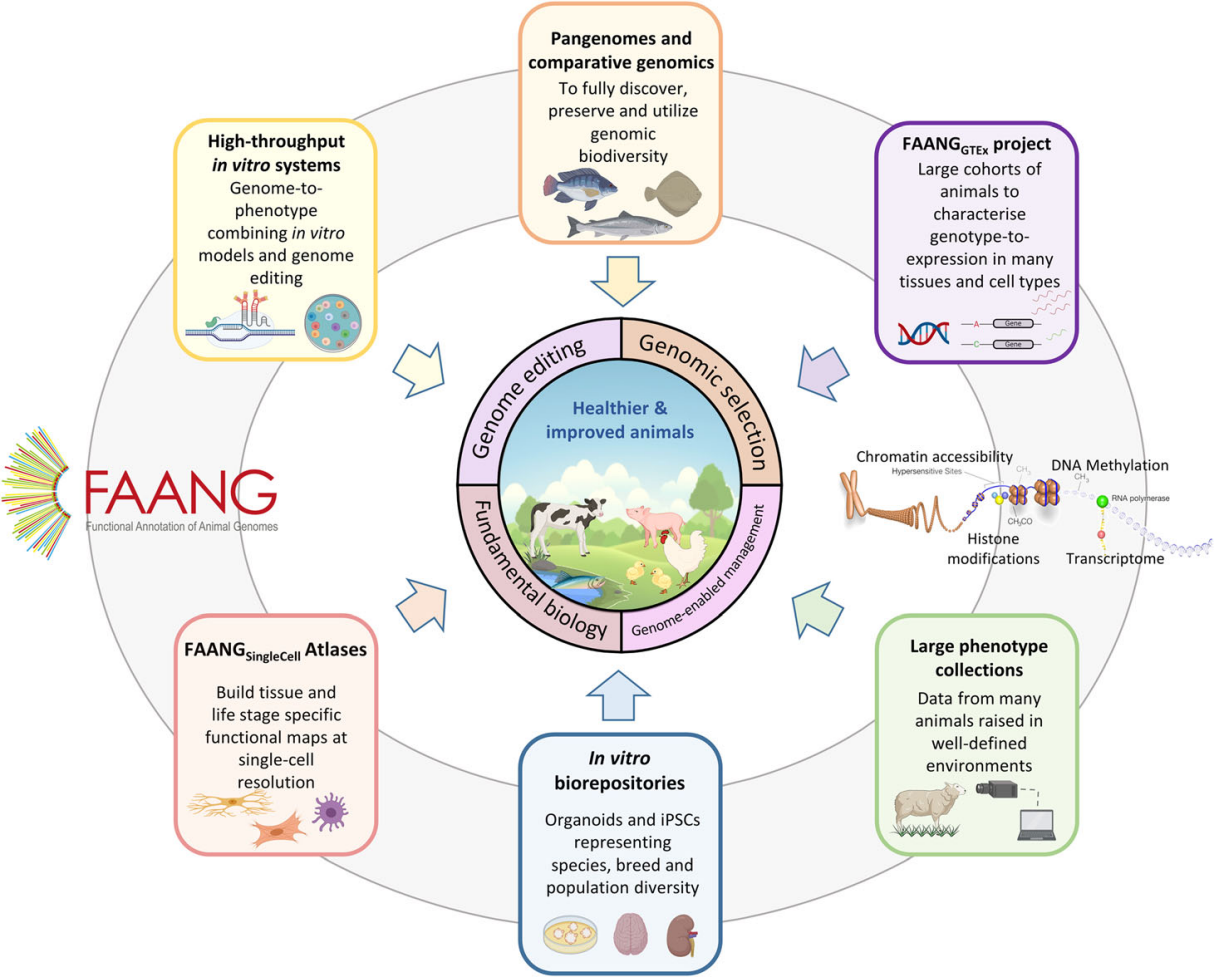
Priorities for the next decade of FAANG research

Here we describe a set of research priorities to meet such present and future challenges that build on progress, successes and resources from the Functional Annotation of ANimal Genomes (FAANG) project [2]. The first stages of FAANG focused on foundational data generation to characterise expressed and regulatory genomic regions, curation and provision of annotated farmed animal genomes [2, 3]. These were largely based on individual level, high depth approaches [3]. The primary challenge facing this community now is harnessing these resources to link genotype, phenotype and genetic merit in order to translate this research out of the laboratory and into industry application in the field. To achieve this effectively, we will need to generate functional genomic information for large populations of animals, rather than relying on a small number of deeply annotated individuals. Furthermore, to date, most of the datasets are from tissues consisting of heterogeneous cell populations, hindering the resolution of functional information and limiting our ability to understand the fundamental cellular and subcellular processes underlying phenotypes. Since the original FAANG white paper was published in 2015 [2], exciting new opportunities have arisen to tackle these challenges. We describe a set of research action priorities for FAANG for the next decade (Fig. 2), in each of the sections below.

## Omics empowered genomic selection

In the past 20 years, genomic selection has substantially increased genetic gain in some farmed animal species through the use of large training populations [4]. However, prediction accuracy in genetically distant populations (i.e. across populations, breeds and generations) remains limited due in part to the current reliance on neutral markers in incomplete linkage disequilibrium with causative genetic variants in the breeding population of interest [4]. Using variants more tightly linked to causative polymorphisms and supported by genomic information in a multi-breed training population can partially alleviate these limitations [5]. Large-scale whole-genome resequencing has produced inventories of many millions of variants for thousands of animals [6]. In such sequence datasets, the causative variants are directly genotyped among millions of neutral markers. This reduces the signal-to-noise ratio when all the data are used for genomic prediction without prior biological information. Efforts to detect causative variants have been successful for variants with large phenotypic effects, often deleterious, using a combination of quantitative, population and molecular genetics [4]. However, economically important traits have a polygenic architecture and causative variants are expected to have small effects, which makes their detection and quantification difficult. Most of these causal variants, with small effects, are likely to be located in regulatory sequences and impact complex traits through changes in gene expression [4]. Thus, it is expected that improvements in prediction accuracy can be achieved by filtering the genetic marker information based upon whether the genetic variants reside in functional sequences and developing robust prediction models that can accommodate the biological priors. As functional (expressed and regulatory) genomic elements are not easy to predict from sequence alone, FAANG will enhance current genome annotation with functional information from a range of relevant tissues, cell types and developmental stages. Recently, novel methods for the integration of biological information (e.g. methylation of regions of predicted functionality) into genomic prediction have been proposed, e.g. [5]. These models, which are based on the combination and ranking of many diverse datasets from multiple animals, could facilitate further improvements in predicting genetic merit and consequently on genomic selection, as has been demonstrated in cattle [5]. As many more suitable datasets will become available in the next 5 years, improving and adapting these methods to enhance genomic prediction accuracy, whilst conserving genetic diversity, across farmed animal species will be a priority for FAANG.

## FAANG_GTEx_—linking genetic variation to genome function

The first phase of FAANG is using a specific set of transcriptomic and epigenomic assays to define functional regions of the genome in tissues [2]. Due to the significant investment per sample, this phase was limited to only a few individuals and ascribed function was averaged across these replicates [2]. Progress has been made in defining functional regions, and this should be built upon to ascertain the effect of genetic variation on genome function [3]. Collecting functional genomic data across many genetically diverse animals lends itself to the application of statistical genomics to detect quantitative trait loci (QTL) controlling molecular phenotypes. This is particularly powerful when done at sequence-level resolution to directly relate molecular phenotypes (e.g. gene expression or methylation information) to variants associated with complex traits. The GTEx consortium (https://gtexportal.org/home/) has achieved this very effectively across human tissues, enabling expression QTL (eQTL) studies linking gene expression to genetic variation [7] and providing a framework for FAANG to develop a similar project for farmed animals (FAANG_GTEx_). Large farmed animal cohorts in controlled and well-characterised environments with extensive pedigree information and molecular phenotypes would allow researchers, in partnership with industry, to (1) build better predictive models of genotype-to-phenotype, (2) better understand genotype-by-environment interactions and (3) prioritise functional variants for inclusion in breeding programmes [4]. Hundreds of thousands of farmed animals currently have imputed genotypes and extended pedigrees with deep phenotypic records [6]. A project analysing the relationship between SNPs from Genome Wide Association Studies and gene expression for cattle, mining publicly available sequence data, was published earlier this year, demonstrating the feasibility, timeliness and potential of a GTEx approach for farmed animals [8].

## Beyond genomic selection: towards genome-enabled management

Beyond its use in genomic prediction, the functional data produced by FAANG will provide new perspectives for informed management decisions. Epigenetic and expression information for individual animals could be combined with microbiome data and high-throughput phenotypes from new management technologies (e.g. wearables, GPS, in-vivo imaging systems) [9]. These datasets from large cohorts of animals would enhance prediction of adaptive capacity at the individual, farm or population level through integration of prior environmental data with individual genome information. Thus, providing new opportunities for informed management decisions during an animal’s lifetime (e.g. to optimise diets or for steering animals into the most appropriate production systems). A genome enabled management approach (providing animals, within a production system, with their specific needs during their lifetime) will be beneficial to improving animal health and welfare, facilitate adaptation to changing environments and contribute to addressing public concerns related to animal production. Achieving this within the next 10 years may be possible, but the challenge will be to ensure it is practical and affordable for animal breeders and producers.

## Understanding and conserving genomic diversity—the power of pangenomes

Through large-scale sequencing efforts by the farmed animal genomics community data are now accumulating that characterise the sequence diversity of farmed animals including locally adapted breeds/populations. As a consequence, future genetic management is likely to include the use of pangenomes that will capture all available population-level genomic information for a given farmed animal species. Using graph-based frameworks, we can more accurately genotype and annotate the genomic diversity present in any given individual [10]. In this way, pangenomes can reveal population- or breed-specific adaptations that could be used to tailor the genotypes chosen in future farming systems in order to conserve biodiversity whilst improving production efficiency and animal health [1]. Furthermore, the highly annotated genomes produced by FAANG allow evolutionary conservation across species to be defined for all genomic features [11]. Ongoing FAANG projects involve comparative analysis which will reveal the functional basis of phenotypes present in one species that are desirable in others. Such projects contribute to addressing the major opportunity that exists to enhance the sustainable production of a wider diversity of animal species, including numerous and diverse aquaculture species that are poised to exploit functional genomics to expedite genetic improvement, where tailored and cost-efficient approaches will be required [12]. Current FAANG-related projects already extend to several major farmed finfish species in Europe and North America. We envisage an increased representation of aquatic species, including shellfish, and further expansion to include invertebrates, within FAANG projects during the next 5 to 10 years.

## FAANG_SingleCell_—deconvoluting transcriptional and regulatory complexity

The use of bulk tissue samples in the FAANG studies performed to date captures regulatory elements and expression signals averaged across all represented cell types but fails to reveal the cell-specific basis of the molecular phenotypes of interest. In order to more accurately link genotype to phenotype, data at the level of individual cell types are required. Single-cell sequencing technologies enable the deconvolution of the transcriptional and regulatory complexity in tissues made up of multiple cell types. New technologies to detect gene expression as well as chromatin accessibility, structure and interactions within single cells provide more comprehensive data to predict function and interaction partners for regulatory elements. As a consequence, one of the main priorities for FAANG within the next 5 to 10 years is to create single-cell atlases for the key tissues of farmed animal species (FAANG_SingleCell_). The organisational processes, standardisation and data sharing infrastructure established by the community for the first stages of FAANG [3] will provide a strong foundation for FAANG_SingleCell_ to progress quickly and efficiently. The FAANG_SingleCell_ project should build on existing functional tissue maps for other species [13] and will enable the identification of genomic variants underpinning trait-linked cell types/factors and causal variants. In the FAANG_GTEx_ project described above, single-cell atlases will provide a powerful layer of resolution including cell-specific molecular phenotypes, enabling the fine-scale dissection of complex traits of interest.

## In vitro systems—bridging the gaps between cell, tissue and whole animal scale knowledge

Single-cell sequencing technologies can also be used to deeply characterise cell and tissue complexity of in vitro systems such as organoids. Over the last 5 years, organoids for many different organ systems and for multiple farmed animal species have been developed [4]. Organoids provide ex vivo/in vitro systems for testing candidate causal variants by genome editing technologies and potentially a system for high-throughput, cost-effective, large-scale in vitro phenotyping. Importantly, given the ease of biobanking, organoids have a strong ethical benefit in reducing the number of animals used in experimentation [3]. Multiple organoid models can be derived from very small quantities of tissue or from induced pluripotent stem cells (iPSCs). They provide the potential to generate and test multiple phenotypes to unravel when, and under what conditions, a putative causal variant has an effect. Therefore, farm animal organoids will be valuable over the coming decade, providing information about fundamental biology to model the effects of changing environmental conditions and supporting immunology, vaccinology, physiology, nutritional and biodiversity conservation studies. The ability to decompose complex phenotypes into key processes will provide a means to robustly relate the deep phenotypes measured in these systems with the traits used for selection, opening to the possibility of using organoids for breeding purposes.

## Genome editing—a route to application for FAANG data

The application of genome editing to farmed animals is advancing rapidly, mainly due to development of CRISPR/Cas technologies [12, 14]. The CRISPR toolbox has expanded to improve precision, allow modulation of gene expression and epigenetic modifications, and now forms an integral part of the future FAANG roadmap [3]. CRISPR-mediated modification of putative genomic elements can confirm their functionality and reveal their roles in cellular (and organoid) function. Genome-wide multiplexed CRISPR approaches now enable the simultaneous interrogation of thousands of genomic features in cell lines, increasing the feasibility of this approach for genome-scale annotation [15]. These high-throughput approaches can also be used in combination with single-cell sequencing technologies to obtain high-resolution molecular phenotypes. In addition, genome editing represents a potential major route for the application of FAANG research in farmed animal breeding programmes via (1) detection and utilisation of causative variants affecting important traits, (2) targeted introgression, or ‘introgression-by-editing’, of favourable alleles from other strains or species into a closed breeding population, or (3) creation of de novo alleles with favourable effects, either predicted from unbiased genome-wide screens or from a priori knowledge of the biology of the trait in question. Public perception and regulatory hurdles remain and ongoing discussion through stakeholder engagement must continue and evolve to keep pace with technological advances. While the use of genome editing for the improvement of farmed animals may currently only be possible in some countries, its use in in vitro models, such as organoids, is not subject to the same legislation and ethical considerations as the use of whole animals and thus represents a new frontier for FAANG research.

## Data recording, computation and integration to support the emerging objectives of FAANG

As a scientific community, FAANG continues to develop a coordinated analysis and data collection infrastructure crucial for its success [3]. The FAANG bioinformatics community, including the centralised Data Coordination Centre (DCC), is focused on open reproducible science, the FAANG data portal (https://data.faang.org/home) is the focal point for this activity. Technological development, coordination and standardisation by the DCC will continue to be crucial for the shift towards population scale studies, single-cell datasets, cell atlases and pangenomes, across a growing number of species. This will require new reproducible analysis pipelines and infrastructure, metadata validation services, data portal features such as a centralised atlas browser and online training resources. Single-cell atlases and in vitro systems for farmed animal species will be accompanied by high quality metadata, archiving and visualisations across species, organ systems, tissues and cell types. As FAANG datasets continue to increase in complexity, there is a growing need for new methods of data visualisation and integration to be made available. These future developments, and the distributed data and analysis infrastructure, will be crucial for the successful application of functional data to farmed animal breeding programmes.

## Priorities for the future of FAANG

**Fig. 3:**
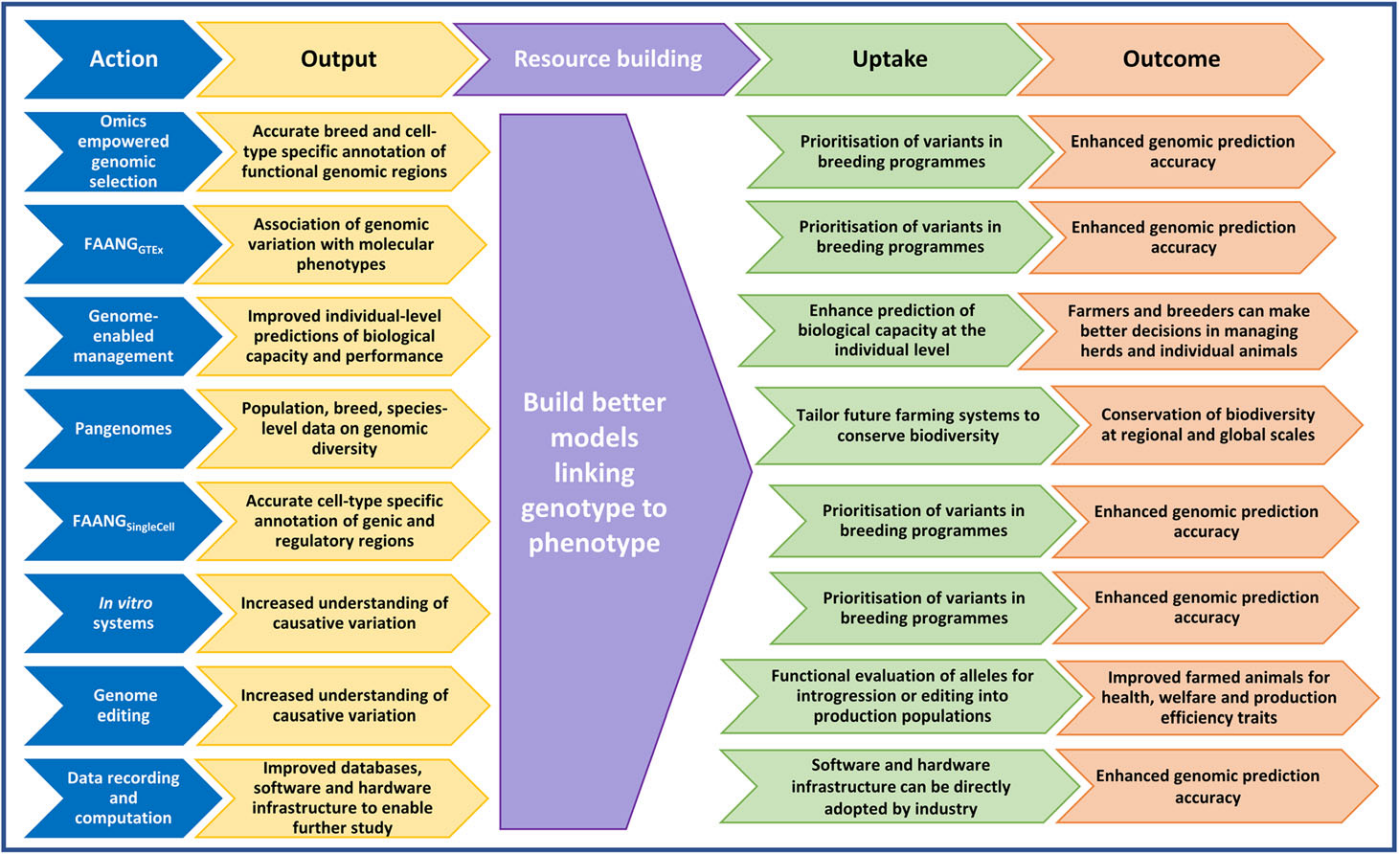
How implementation of FAANG research priorities over the next decade will benefit farmed animal production

The research priorities we have outlined for FAANG for the coming decade are depicted in Fig. 2. The uptake by the farmed animal production industry and the expected outcomes of each prioritised action are summarised in Fig. 3. FAANG will improve our ability to more accurately use genotype to predict phenotype. This will directly contribute to addressing the challenges faced for sustainable and responsible global food production in the next decade (Fig. 1). However, whilst the molecular assays used to enable functional annotation can now be delivered at much lower cost, the costs for the research priorities outlined above remain substantial, especially considering the rapid increase in number and diversity of target species in the aquaculture sector. As such, a strong commitment to invest in research is needed. Persuading the US Department of Agriculture and the European Commission to include FAANG projects in NIFA-AFRI and Horizon 2020 funding calls, respectively (https://faang.org/proj.php) was a major success for the first stage of FAANG and its leadership. Current funding for FAANG supports the research community to improve the functional annotation of key farmed animal species and to facilitate more refined genomics-enabled animal breeding/genetic improvement. The research priorities outlined here are already strategically aligned to the objectives of the European Green Deal (https://ec.europa.eu/info/strategy/priorities-2019-2024/european-green-deal_en) and current USDA National Institute for Food and Agriculture programmes (e.g. https://nifa.usda.gov/program/genome-phenome-initiative; https://www.ag2pi.org). International cooperation will be essential to secure funding for their achievement. Given the scale and cost of the research involved, it will likely be necessary to initially prioritise the development of in vitro systems and the enhancement of data infrastructure to provide a solid foundation for FAANG_SingleCell_ and FAANG_GTEx_.

The timely achievement of all of the research priorities we outline here for the next stages of FAANG will together increase the capacity of the farmed animal production industry to face the challenges of the future, empowering genomic selection, enhancing adaptation to changing environments, conserving biodiversity and bridging the gaps between cellular and whole animal scale knowledge.
